# Intelligent extraction of reservoir dispatching information integrating large language model and structured prompts

**DOI:** 10.1038/s41598-024-64954-0

**Published:** 2024-06-19

**Authors:** Yangrui Yang, Sisi Chen, Yaping Zhu, Xuemei Liu, Wei Ma, Ling Feng

**Affiliations:** 1 School of Information Engineering, North China University of Water Resources and Electric Power, Zhengzhou, 450000 Henan China; 2 Collaborative Innovation Center for Efficient Utilization of Water Resources, Zhengzhou, 450000 Henan China

**Keywords:** Reservoir dispatching procedures, Information extraction technology, Large language model, Structured prompt language, AI agent, Hydrology, Engineering

## Abstract

Reservoir dispatching regulations are a crucial basis for reservoir operation, and using information extraction technology to extract entities and relationships from heterogeneous texts to form triples can provide structured knowledge support for professionals in making dispatch decisions and intelligent recommendations. Current information extraction technologies require manual data labeling, consuming a significant amount of time. As the number of dispatch rules increases, this method cannot meet the need for timely generation of dispatch plans during emergency flood control periods. Furthermore, utilizing natural language prompts to guide large language models in completing reservoir dispatch extraction tasks also presents challenges of cognitive load and instability in model output. Therefore, this paper proposes an entity and relationship extraction method for reservoir dispatch based on structured prompt language. Initially, a variety of labels are refined according to the extraction tasks, then organized and defined using the Backus–Naur Form (BNF) to create a structured format, thus better guiding large language models in the extraction work. Moreover, an AI agent based on this method has been developed to facilitate operation by dispatch professionals, allowing for the quick acquisition of structured data. Experimental verification has shown that, in the task of extracting entities and relationships for reservoir dispatch, this AI agent not only effectively reduces cognitive burden and the impact of instability in model output but also demonstrates high extraction performance (with F1 scores for extracting entities and relationships both above 80%), offering a new solution approach for knowledge extraction tasks in other water resource fields.

## Introduction

Reservoir dispatching regulations are foundational documents for reservoir operation^1^. Water resource departments must strictly follow these regulations during operation to ensure the safety of hydraulic hub projects, thereby fully realizing the comprehensive benefits of flood control, power generation, navigation, and water resource utilization^2^. However, reservoir dispatching regulations are often written in natural language, lacking structure, which means complex entities and relationships are embedded within the text. For example, the dispatching text "In the event of a major flood in the upper Yangtze River, when the water level of the Three Gorges Reservoir is between 171 and 175 m, discharge must be controlled to ensure that the water level at Shashi does not exceed 45 m" expresses that with "the water level of the Three Gorges Reservoir at 171–175 m", the method of "controlling discharge" is adopted, and "controlling discharge" can achieve the dispatching goal of "keeping Shashi water level no higher than 45 m". Another dispatching text "Flood control dispatching mainly involves flood compensation for the Jingjiang river section" shows that "flood control dispatching" involves "flood compensation for the Jingjiang river section" as a method of dispatching. Once these entities and relationships embedded in the text are extracted using information extraction technology, they can be organized into triples^3^, thereby providing structured knowledge support for tasks such as generating reservoir dispatching plans and digital twins^4^.

As the infrastructure of water conservancy projects continues to improve, enhancing flood disaster prevention capabilities, it also brings increasingly complex project dispatching rules and basin dispatching issues. To quickly and efficiently extract high-value information and assist decision-makers in precise reservoir dispatching, researchers in the field are using natural language prompts to guide large language models to perform specific downstream tasks,and to develop applications based on this approach. This method is considered a new form—Artificial Intelligence agent(AI agent)^5^, which solve problems by defining inputs and outputs and using natural language prompts as a medium to call upon the computational power of large language models^6^. Although the operation is convenient, the AI agents overly rely on designed natural language prompts and lack precise methodologies for controlling large language models^7^. Therefore, when dealing with complex application scenarios such as reservoir dispatching, it is often difficult to ensure output stability. There are two major challenges for intelligent agents when extracting entities and relationships in the field of reservoir dispatching. first, cognitive burden^8–10^: In the field of reservoir dispatching, there is a large number of specialized terminology. Without explanation, large language models are prone to overlook the domain-specific knowledge background when interpreting natural language prompts, leading to semantic ambiguity and subsequent extraction errors. For example, given the prompt "Flood control dispatching mainly involves flood compensation for the Jingjiang River section. When the water level of the Three Gorges Reservoir is between 171 and 175 m, control the flow at Zhicheng Station to ensure that the water level at Shashi Station does not exceed 45.0 m. Please identify the dispatching requirements, dispatching mode, preconditions, dispatching measures, and dispatching goal contained in the above text," the large language model, due to the lack of domain-specific knowledge, might ignore the entity "flood compensation for the Jingjiang River section" under the category of " dispatching mode.". Second, instability in model output^11–13^: Due to the fact that large language models are trained on information from various domains and their internal decision-making pathways are extremely complex, facing complex reservoir dispatch texts, In the absence of constraints, even using the same natural language prompt can lead to the model generating outputs through different pathways, thereby affecting the stability of the output results. Furthermore, the inherent black-box nature of large language models^14^ also increases the difficulty of precise control, making it challenging to trace and diagnose unexpected outputs.

To reduce the cognitive burden and output instability of large language models, this paper proposes a method for guiding large language models in entity and relationship extraction using structured prompt language. This method precisely conveys the requirements through structured prompts while establishing rules to ensure the prompts are both constrained and effective. Additionally, based on this method, an AI agent is designed using software engineering principles to facilitate use by reservoir dispatching professionals. Specifically, this paper refines eight standardized labels from existing prompt design patterns^15–17^ based on task scenarios, covering essential content for extracting reservoir dispatching entities and relationships. The labels are presented using symbols such as @, {}, and indentation to display their hierarchical structure. Alleviating its cognitive burden and output instability by providing the large language model with comprehensive requirements, supplying restrictive rules, explaining the specific meanings of entity and relationship types, and offering extraction examples. The eight labels and their hierarchical structure are organized and completed using BNF^18–20^ for grammar definition, forming a complete structured format that reduces semantic ambiguity and facilitates model understanding, thereby improving output accuracy. Each label is further described with relevant reservoir dispatching procedures, assigning them specific functions to accomplish the entity-relationship extraction task. Finally, leveraging software engineering design principles, an intelligent agent is developed based on the structured prompt language to aid reservoir dispatching personnel. The method and developed AI agent described in this article can assist dispatching professionals in quickly obtaining reservoir dispatching-related entities and relationships, laying a solid foundation for the timely generation of dispatching plans. This approach breaks the cognitive burden and output instability caused by unclear expressions, non-standard writing, and the lack of restrictions when using natural language prompts. This new paradigm of knowledge extraction provides a fresh perspective for various information extraction tasks. In the future, it can also be applied to other fields of water conservancy, promoting the digital and intelligent development of text processing in water conservancy engineering to a new stage.

The main contributions of this paper are as follows:

1. This paper proposes a reservoir dispatching entity relationship extraction method based on structured prompt language. This method refines eight types of labels based on extraction task scenarios, effectively covering all key aspects of creating prompts for reservoir dispatching entity relationship extraction. By creating comprehensive and high-quality prompts, establishing restrictive rules, and providing cases, the method alleviates the cognitive burden and output instability problems of large language models.
2. The proposed method uses the BNF paradigm to combine labels and their hierarchical structures to form a fixed structured format. This makes the overall content more intuitive and easier for large language models to understand, ensuring the standardization and effectiveness of prompt design. It enables large language models to more efficiently extract reservoir dispatching entities and relationships from the corresponding texts, laying the foundation for the rapid generation of reservoir dispatching plans.
3. By leveraging software engineering design principles, this paper develops an AI agent based on structured prompt language for reservoir dispatching entities and relationships. This agent is easy for dispatching practitioners to operate; they only need to input the text to obtain the corresponding entities and relationships. The AI agent automates the extraction process for reservoir dispatching practitioners, accelerating the structured processing of reservoir dispatching text entities and relationships.

## Related work

In the field of water conservancy, extracting text information has always been a research focus. Existing studies mainly concentrate on using model training methods for extraction work. For example, Zou et al. combined Word2vec and TFIDF for extracting features from water conservancy engineering quality supervision texts^21^. Wang et al. integrated PTM and MCNN to extract triplets from water conservancy engineering emergency plans^22^. Yang et al. constructed a joint extraction framework based on T5^23^ and BERT^24^ to identify relevant entities in the water conservancy discipline^25^. However, these model training-based extraction methods require manual data annotation, which consumes a significant amount of time. As more engineering dispatching rules emerge and watershed dispatching problems become increasingly complex, model-based extraction cannot effectively achieve the goal of timely generating reservoir dispatching plans during emergency flood control periods. With the advent of large language models (such as GPT-3^13^), scholars have begun using natural language prompts to guide these models in performing specific downstream tasks, leading to the design of intelligent agent applications. Due to their simplicity of operation and the powerful computational capabilities of large language models, these applications have been widely used in various fields. For example, IBM Watson Health^26^ analyzes large amounts of data in the medical field to provide treatment recommendations; Minedojo^27^ offers thousands of tasks in the gaming field, allowing people to freely explore a 3D world; and Amazon's Alexa^28^ optimizes supply chain management in the retail industry through personalized recommendations. The reason intelligent agents perform well in these fields is that large language models can understand simple natural language prompts. However, when dealing with complex application scenarios such as reservoir dispatching, they face cognitive burden and output instability. To alleviate these two phenomena, Sewon et al.^29^ began experimenting with presenting information using bullet points or dividing it into different parts to more clearly describe relevant prompts, helping users with expression. Xing et al.^30^ proposed a guiding prompt technique, suggesting the formulation of certain constraints and providing case references to mitigate the effects of the inherent black-box nature of large language models, thereby improving output stability. Inspired by the above content, this paper proposes a method for guiding large language models in entity and relationship extraction using structured prompt language. Additionally, based on this method, an intelligent agent was designed using software engineering principles to assist reservoir dispatching professionals.

### Ethics statement

This study was conducted in accordance with the Declaration of Helsinki and relevant national laws and regulations. All experimental methods have been approved by the Ethics Review Committee of North China University of Water Resources and Electric Power to ensure compliance with internationally recognized ethical standards. All participants were volunteers, and their anonymity and confidentiality were guaranteed.

## Construction and application of structured prompt language

### Domain knowledge modeling

**Figure 1:**
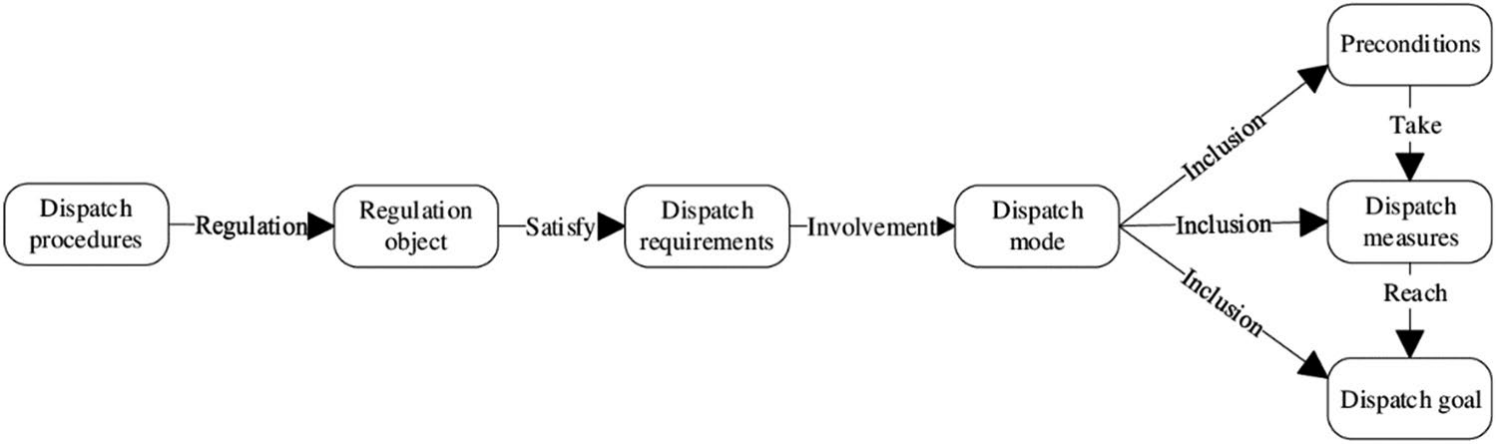
Design diagram of the ontology framework.

To better extract triples closely related to reservoir dispatch and thereby facilitate applications in this field, it is first necessary to define the entities and types of relationships contained within reservoir dispatch texts, completing the design of the entity-relationship ontology framework to make the extraction targets more focused. Therefore, through in-depth analysis of related textual data, this paper summarizes 7 types of entities and 6 types of relationships as subjects for experimentation. The entity types include dispatch procedures, regulation object, dispatch requirements, dispatch mode, preconditions, dispatch measures, and dispatch goal; the relationship types include regulation, satisfy, involvement, inclusion,take, and reach. The design of the ontology framework is shown in Fig. 1.

### labels

**Figure 2:**
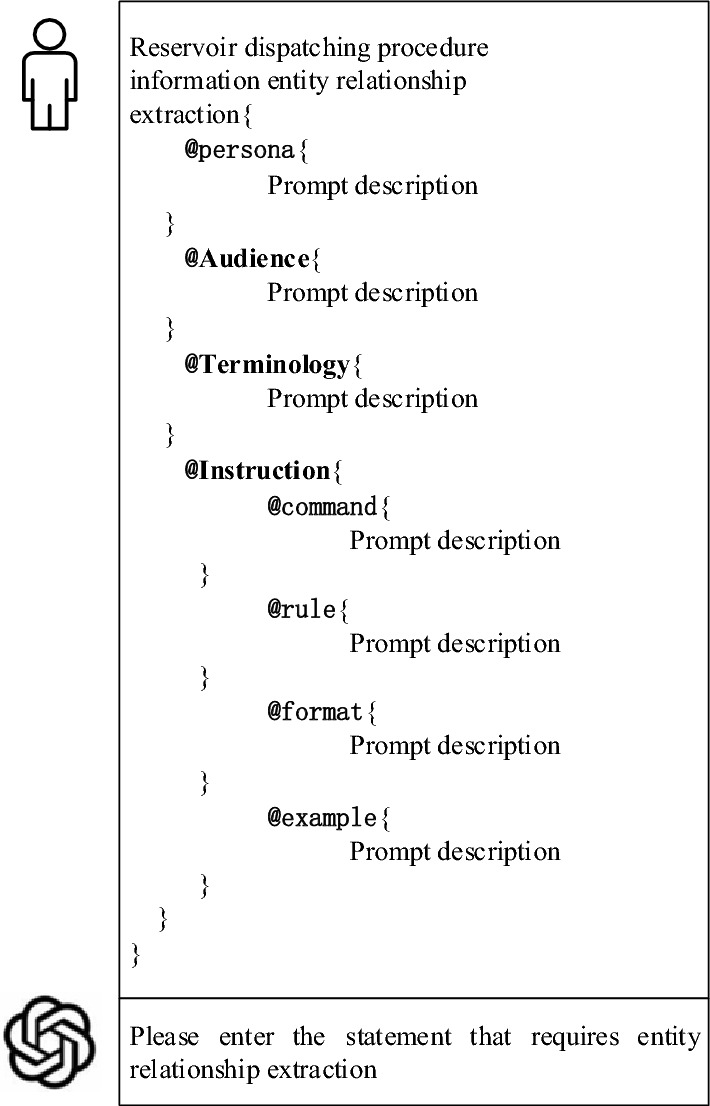
Complete structure of structured prompt language.

The structured prompt language comprises 8 labels, among which "Persona", "Audience", "Terminology", and "Instruction" are important labels, while "Rule", "Command", "Format", and "Example" serve as supporting labels. Among these eight labels, "Persona" informs the large language model of the persona it should adopt, enabling it to provide comprehensive knowledge in the field of reservoir dispatching; "Audience" informs the model that the target audience is industry professionals, ensuring its responses are standard and easy to understand; "Terminology" provides the specific meanings of specialized terms in the field of reservoir dispatching; "Instruction" specifies the next task, such as waiting for input from reservoir dispatching professionals; "Command" instructs the large language model to execute the extraction task; "Rule" informs the model of the complex dispatching rules in the field of reservoir dispatching and the considerations it must take into account; "Format" specifies the desired format of the answers; "Example" guides the model to understand the embedded entities and relationship patterns in the given example to perform the task. This paper utilizes the BNF to organize the labels and define their syntax, completing the construction of the structured prompt language. The complete structural framework of the structured prompt language is shown in Fig. 2.

#### “Persona” and “Audience”

**Figure 3:**
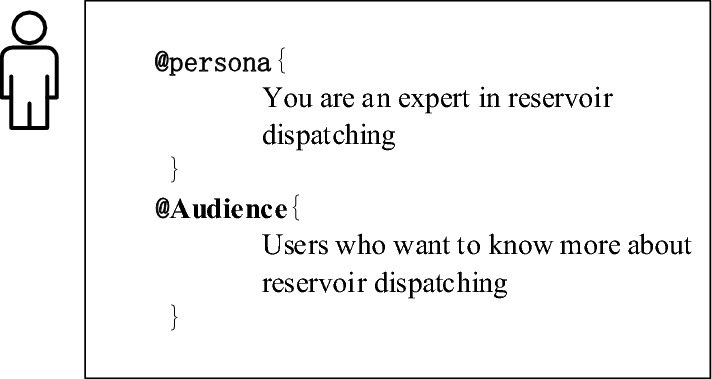
Specific task descriptions of persona and audience.

To enable large language models to provide comprehensive data support and decision analysis from the perspective of the reservoir dispatch field, assisting reservoir management personnel in achieving rational dispatching and optimized utilization of water resources, this paper designates the large language model to play the role of "an expert in the field of reservoir dispatch," allowing it to role-play to better accomplish specific tasks. Hence, the label "Persona" is defined in this context. Moreover, to ensure that the large language model's responses are more standard and understandable to professionals in the reservoir dispatch field, the label "Audience" is derived by extending the "Persona" label. The BNF is used to organize and define its grammar, which can be represented as " < Persona Part > :: =  < @Persona > {Persona Prompt Description}"; " < Audience Part > :: =  < @Audience > {Audience Prompt Description}". The specific content related to "Persona" and "Audience" in the task of extracting information from reservoir dispatch regulations is shown in Fig. 3.

#### “Terminology”

**Figure 4:**
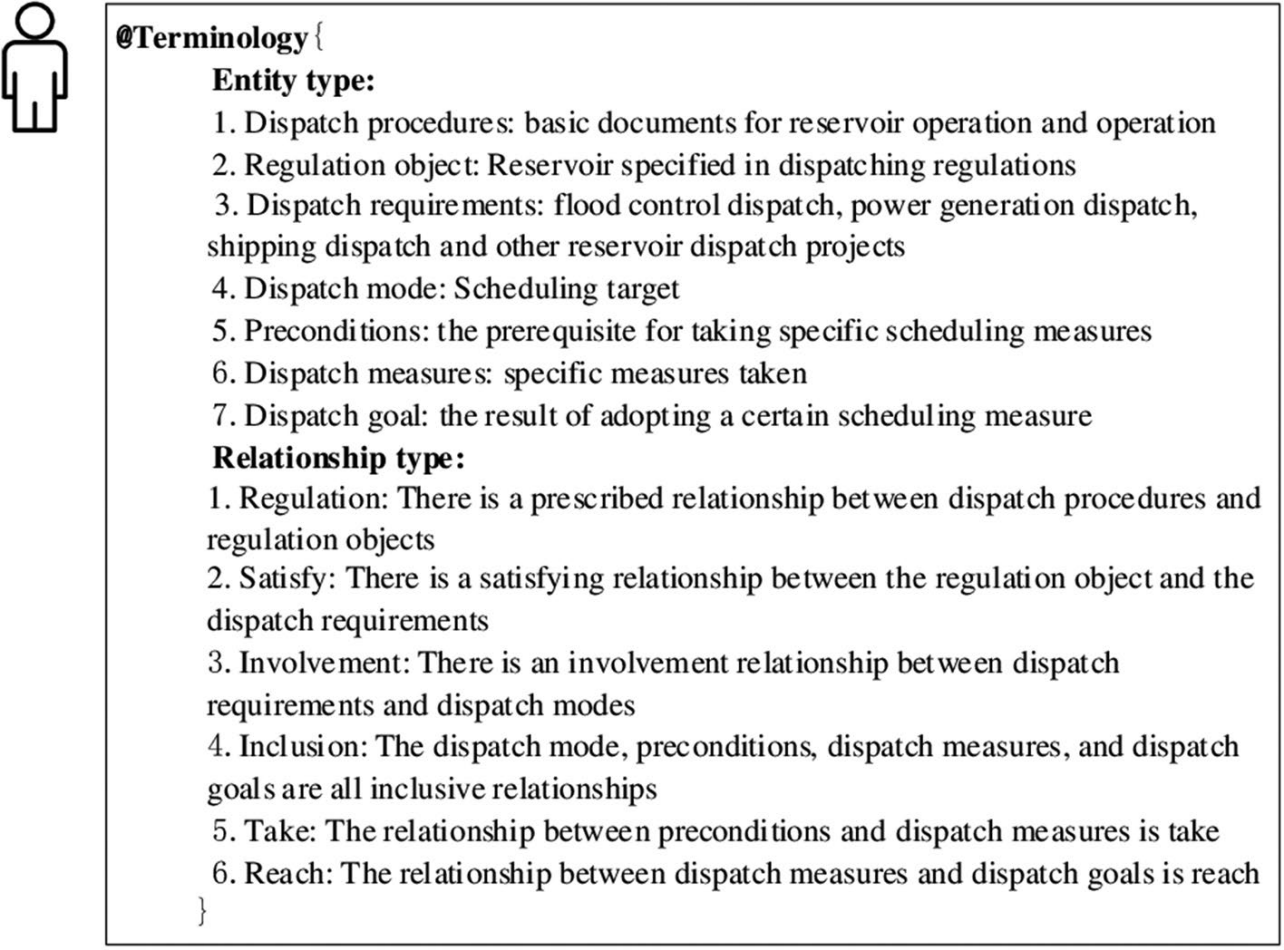
Specific content of terminology.

Due to the presence of a large number of professional terminologies in the reservoir dispatch field, which can lead to misunderstandings, for example, the entity type "dispatch measures" required by this paper is understood in its literal sense as methods adopted in response to different hazardous scenarios. However, in the field of reservoir dispatch, "dispatch measures" specifically refer to the methods involved in dispatch mode, while solutions appearing in dispatch requirements are considered dispatch mode. In the process of formulating task prompts, it is necessary to clarify relevant terms in the reservoir dispatch field to help the large language model better understand the content encompassed by the terms. Consequently, this paper identifies the label "Terminology", which is organized and defined using the BNF, and can be represented as " < Terminology Part > :: =  < @Terminology > {Terminology Prompt Description}". Furthermore, it describes the specific content of "Terminology", i.e., the entity and relationship types involved in the task of extracting entity relationships from reservoir dispatch regulations, as shown in Fig. 4. It's worth noting that, fundamentally, "Terminology" serves as a means of establishing a common language with the large language model, ensuring it understands the meanings of related types through clear explanations to execute specific tasks.

#### “Instructions” and supporting labels

**Figure 5:**
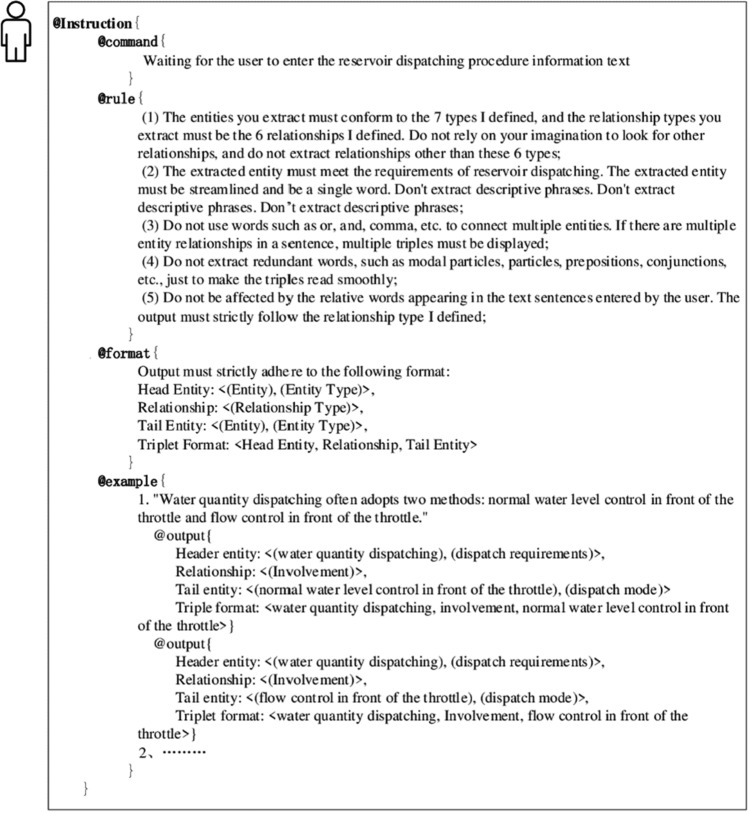
Specific content of the instruction.

Due to its extensive world knowledge, large language models are prone to introducing noise from other domains when tasked with text extraction in the field of reservoir dispatch. To emphasize that the task it performs should align with the "Persona", this paper refines the " Instruction" label. Moreover, to constrain the "Instruction", the paper further extends four labels: "Rule", "Command", "Format", and "Example", creating a certain hierarchy with " Instruction" to better control the phenomenon of unstable outputs from the large language model. Among these, "Command" is utilized to help complete sub-steps of the "Instruction", mainly to issue instructions to the large language model, informing it of what it initially needs to do; "Rule" strongly constrain the "Instruction". In this paper, to better complete the overall task, through continuous testing and verification, five related "Rule" were ultimately formulated based on the reservoir dispatch regulation information extraction task scenario to regulate the behavior of the large language model. These mainly instruct the large language model to emphasize its own rules, directing it to carry out the overall task according to the rules established by this paper, preventing the introduction of noise from other domains by the large language model. Furthermore, due to the complexity of entity objects in the field of reservoir dispatch, where it is common to connect entity objects using conjunctions, for example, the text "Water volume dispatch often uses normal water level control before the throttle gate and flow control before the throttle gate methods for processing" might result in the extraction of only one entity "normal water level control before the throttle gate and flow control before the throttle gate methods" during the extraction process. Therefore, it is necessary to inform the large language model of this issue and impose rule constraints on it to address the extraction irregularities caused by words expressing semantic parallelism or progression, such as "or", "and", and commas. "Format" instructs the large language model to output in a fixed structure of triples; "Example" consists of sentences from dispatch texts and their extraction results, serving as examples for the large language model to learn from. This helps it understand how to extract the entities and relationships contained within the examples, to better meet the task requirements of this paper and adhere more closely to the specified format. When selecting examples, priority is given to sentences that cover a broader range of water management dispatch information and are semantically more complex. The BNF is used to organize and define the syntax for the aforementioned labels, which can be represented as " < Instruction Part > :: =  < @Instruction >  < Instruction Prompt Description Part > "; the Instruction Prompt Description Part is further detailed as " < Instruction Prompt Description Part > :: =  < Rule Part >  < Command Part >  < Format Part >  < Example Part > ", where" < Rule Part > :: =  < @Rule > {Rule Prompt Description}; < Command Part > :: =  < @Command > {Command Prompt Description}; < Format Part > :: =  < @Format > {Format Prompt Description}; < Example Part > :: =  < @Example > {Example Prompt Description}". The specific prompt descriptions for "Command", "Rule", "Format", and "Example" are shown in Fig. 5.

### AI agent

**Figure 6:**
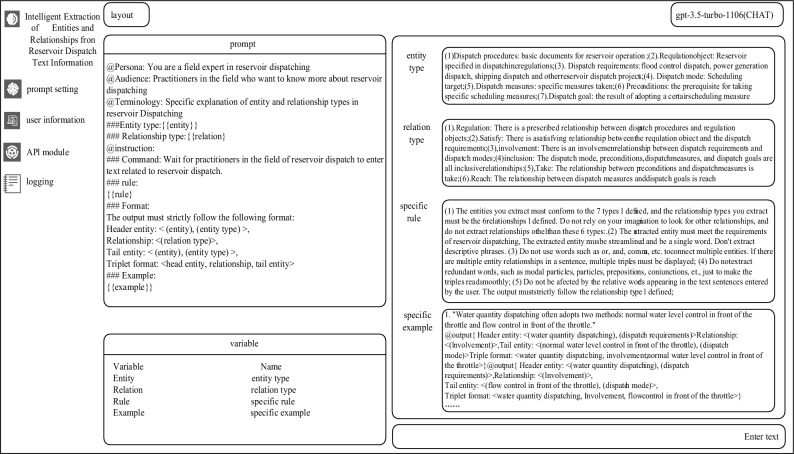
Prompt setting page diagram.

To facilitate the operation by dispatch personnel and swiftly complete the task of extracting entity relationships from reservoir dispatch texts, this paper develops an AI agent based on structured prompt language, utilizing software engineering design principles. This AI agent comprises four main modules: the Prompt Setting module, User Information module, API module, and Logging module. The Prompt Setting module is primarily used for the execution and management of the process of extracting entities and relationships from reservoir dispatch regulation texts, including establishing the entity and relationship extraction tasks, selecting the appropriate large language model (e.g., GPT-3.5^31^, GPT-4^32^, etc.) based on specific conditions, uploading reservoir dispatch texts, and outputting the results of entity and relationship extraction. The User Information module stores user data, password settings, and system logins. The API module keeps the API-KEYs required to call various large language models. The Logging module documents the user's operation process and the results outputted. The specific page for the prompt setting is shown in Fig. 6. This AI agent enables reservoir dispatch personnel to achieve automated extraction using large language models based on structured prompt language, speeding up the process of structuring the entities and relationships in reservoir dispatch texts.

### Extract case display

Taking the Three Gorges-Gezhouba Water Conservancy Project dispatching procedure text as the extraction object, some triplets obtained by using the agent to extract reservoir dispatching information entities and relationships will be displayed. Specific triplet examples are shown in Table 1.

**Table 1:** shows examples of triples.

Relationship type	Extract instances
Regulation	(《Preliminary design report of Yangtze River Gezhouba Water Conservancy Project》, regulation, Gezhouba Water Conservancy Project) (《Preliminary design report of the Yangtze Three Gorges Water Conservancy Project》, regulation, Gezhouba Water Conservancy Project) (《Preliminary design report of the Yangtze Three Gorges Water Conservancy Project》, regulation, Sanxia Water Conservancy Project) ……
Satisfy	(Gezhouba Water Conservancy Project, satisfy, Flood control dispatch) (Gezhouba Water Conservancy Project, satisfy, Generation dispatch) (Gezhouba Water Conservancy Project, satisfy, shipping dispatch)……
Involvement	(Flood control dispatch, involvement, Flood control compensation for the Jingjiang River section) (Flood control dispatch, involvement, Flood control compensation for Chenglingji area) (Generation dispatch, involvement, The use of the main stream in the Three Gorges Reservoir area and the downstream channel of Gezhouba Dam)……
Inclusion	(Flood control compensation for the Jingjiang River section, inclusion, The water level of the Three Gorges Reservoir is between 171 and 175 m) (Flood control compensation for the Jingjiang River section, inclusion, Controlling and compensating the traffic at Zhicheng Station) (Flood control compensation for the Jingjiang River section, inclusion, The water level at Shashi Station is not higher than 45.0 m)……
Take	(The water level of the Three Gorges Reservoir is between 171 and 175 m, take, Controlling and compensating the traffic at Zhicheng Station) (The water level in Shashi reaches or exceeds 44.5 m, take, Controlling reservoir discharge flow) (The reservoir water level is the flood control limit level, take, The discharge amount is equal to the incoming amount)……
Reach	(Controlling and compensating the traffic at Zhicheng Station, reach, The water level at Shashi Station is not higher than 45.0 m) (Controlling reservoir discharge flow, reach, The water level at Shashi Station is not higher than 44.5 m) (The discharge amount is equal to the incoming amount, reach, Keep the reservoir water level at the flood control limit level)……

## Experimental settings

This study required human participants for method performance evaluation. Although it did not involve clinical trials, the study was conducted in accordance with the Declaration of Helsinki and relevant national laws and regulations. All experimental methods were approved by the Ethics Review Committee of North China University of Water Resources and Electric Power, ensuring compliance with internationally recognized ethical standards. All participants provided informed consent prior to their participation in the study. The anonymity and confidentiality of the participants were guaranteed, participation was entirely voluntary, and there were no conflicts of interest.

### Data preparation

To verify whether the AI agent can effectively reduce the cognitive burden and the impact of model output instability, and to test its performance in extracting entity relationships from reservoir dispatch regulation texts, this paper employs web crawling technology to gather data. Additionally, it collects dispatch guidelines for reservoir operations, water supply dispatch plans, and similar texts from various water-related departments, resulting in nearly 25 sets of dispatch regulation texts from different reservoirs. Redundant text was processed to remove descriptions unrelated to reservoir dispatching. Then, the filtered paragraphs were segmented into sentences using the natural language processing tool PyLTP, with periods as separators, resulting in 4261 text segments. Professional experts in water resources dispatching were sought to annotate the obtained data according to the entity types and relationship types defined in Section "Domain knowledge modeling". The annotated data served as the benchmark for evaluating the reservoir dispatching text entity relationship extraction results of the AI agent using natural language prompts.

### Baseline experiment setup

#### Extract performance tests

To evaluate the performance of the AI agent designed based on structured prompt language and traditional natural language prompts in extracting entities and relationships from reservoir dispatching procedure texts, this study will invite five graduate students in water resources with at least two weeks of experience using large language models, ensuring they have the capability to effectively create natural language prompts. First, the graduate students will be asked to collaboratively write corresponding natural language prompts based on the reservoir dispatching entity relationship extraction task. Next, the reservoir dispatching procedure data collected in Section "Data preparation" will be used to allow both the natural language prompts and the intelligent agent to extract entities and relationships. The extracted triples will then be compared with the benchmark data. The experimental evaluation will compare the performance of both methods in extracting entities and relationships, using precision (P), recall (R), and F1 score^33^ as the standards. Here, P is the ratio of the number of correctly identified entity relationships to the total number of identified entity relationships, as shown in Eq. (1); R is the ratio of the number of correctly identified entity relationships to the total actual number of entity relationships contained in the text, as shown in Eq. (2); the F1 score is the harmonic mean of precision and recall, as shown in Eq. (3).

1 $$P= \frac{TP}{TP+ FP}\times 100\%$$

2 $$R= \frac{TP}{TP+ FN}\times 100\%$$

3 $$F1= \frac{2 }{TP+ FN}\times 100\%$$

#### Ablation experiment

To verify that the AI agent designed based on structured prompt language can alleviate cognitive burden and the impact of model output instability, this paper will conduct five ablation experiments. The first four ablation experiments will each remove one important label to verify that these labels can effectively alleviate the impact of cognitive load and output instability. The fifth ablation experiment will remove bullet points, label names, and the overall structured format. In this experiment, the structured prompts formulated in this paper will be directly converted into natural language prompts with the same meaning based on the content. These will then be input into the large language model for performance testing. This will verify that dividing the overall content into multiple parts using bullet points, label names, and an overall structured format makes it easier for the large language model to understand, thereby effectively improving accuracy. The experimental standards and methods used are the same as those in Section “Extract performance tests”.

#### Overall effect verification

This paper will verify the overall reading effectiveness of the AI agent from three aspects: logic, relevance, and readability^34^. Logic is primarily used to evaluate the rationality of logic. The assessment of logic in this paper will be conducted from three perspectives: the coherence of the context, the consistency of sentence content with common sense, and the absence of logical issues such as repetition or ambiguous expressions. Relevance mainly measures how well the prompts designed in this paper relate to the overall task and whether they can meet the requirements of the task of extracting entity relationships from reservoir dispatch regulations. Readability assesses whether participants can accurately understand each label and its specific content. This paper will use a three-point system to score logical consistency, relevance, and readability, where higher scores reflect a positive attitude towards the performance of the tool, and lower scores indicate a more negative attitude. Participants will be invited to rate the AI agent developed for extracting entity relationships according to reservoir dispatch regulations.

## Experimental results

### Extract performance test results

The performance test results of the AI agent and natural language prompts for extracting texts from reservoir dispatch regulations are shown in Table 2. The results indicate that the F1 scores for extracting entities and relationships using natural language prompts are 0.629 and 0.652, respectively, while the F1 scores for extracting entities and relationships using the AI agent are 0.797 and 0.802, respectively. This represents an increase of 26.7% and 23.0%, respectively. The reason for this phenomenon is that the structured language prompts used by the intelligent agent have refined eight types of labels based on existing prompt patterns and combined with the extraction task scenarios in the reservoir dispatching field. These labels cover all important conditions in the reservoir dispatching domain, including specific explanations of entity relationship types, restrictive rules, and extraction cases. Bousselham et al.^35^ tested named entity tasks on large language models using domain data, but the extraction results were poor due to the lack of a few samples in the natural language prompts for the large language model to reference. García-Barragán et al.^36^ mentioned that when performing entity extraction using large language models, listing entity type definitions in the natural language prompts and providing corresponding explanations to prove their compatibility can control the output of the large language models to some extent. Additionally, providing a few extraction entity samples to the large language model and using these samples as references to perform tasks on new input data can improve extraction results. The method proposed in this paper includes the aforementioned content to improve extraction accuracy. For example, the "example" label provides extraction examples, the "rule" label sets a series of restrictive conditions for the large language model, In addition, there are other labels that explain specialized terms and constrain the large language model. According to the data in Table 2, this method can effectively improve extraction accuracy, resulting in significant increases in the F1 scores for entity and relationship extraction.

**Table 2:** Extract performance test results.

Type	Entity			Relationship		
	P	R	F1	P	R	F1
Natural language prompt	0.652	0.608	0.629	0.634	0.642	0.652
AI agent	0.812	0.789	0.797	0.815	0.798	0.802

### Ablation experiment results

The results of the ablation experiment are shown in Table 3. From the table, The F1 scores for entity and relationship extraction decreased by approximately 10% compared to the AI agent when the persona and audience labels were removed. This is because defining persona and audience can limit the generation range of the large language model, preventing the generation of irrelevant text and effectively improving the quality and relevance of the output content. Removing these labels introduced noise from other fields, resulting in a decline in the F1 scores for entity and relationship extraction.The F1 scores for entity and relationship extraction decreased by over 23% compared to the AI agent when the terminology and instruction labels were removed. This is because terminology and instruction provide explanations of relevant nouns, extraction cases, and various rule constraints in the reservoir dispatching field for the large language model. Kaushik et al.^37^ and Kanwal^38^ have previously proposed that this approach can effectively constrain large language models and reduce the instability of outputs. After removing these two important labels, the large language model lacks specialized domain knowledge and constraints, leading to a significant decrease in the F1 scores for entity and relationship extraction.The F1 scores for entity and relationship extraction decreased by 6.7% and 9.0% respectively compared to the AI agent when bullet points, labels, and structured forms were removed. This is because bullet points, labels, and structured forms can divide the overall content into different parts to describe related tasks more clearly and intuitively present prompt content. Sewon et al.^29^proposed that dividing the overall content into multiple parts can help large language models better understand the context and perform better, and Singh et al.^16^indicated that structured forms similar to programming languages are easier for large language models to understand. Therefore, removing bullet points, labels, and structured forms can cause large language models to misunderstand, resulting in a decline in the F1 scores for entity and relationship extraction. In conclusion, the results of five ablation experiments demonstrate that the labels, content, bullet points, labels, and structured forms contained in the AI agent can effectively reduce the cognitive burden and the impact of output instability on large language models.

**Table 3:** Ablation experiment test results.

Type	Entity			Relationship		
	P	R	F1	P	R	F1
Remove "persona"	0.759	0.733	0.746	0.732	0.726	0.729
Remove "audience"	0.763	0.746	0.756	0.762	0.752	0.749
Remove "terminology"	0.656	0.645	0.651	0.612	0.621	0.615
Remove "instruction"	0.632	0.643	0.646	0.628	0.616	0.634
Remove " bullet points, labels, and structured forms "	0.755	0.741	0.747	0.744	0.732	0.736
AI agent	0.812	0.789	0.797	0.815	0.798	0.802

### Overall effect verification results

**Figure 7:**
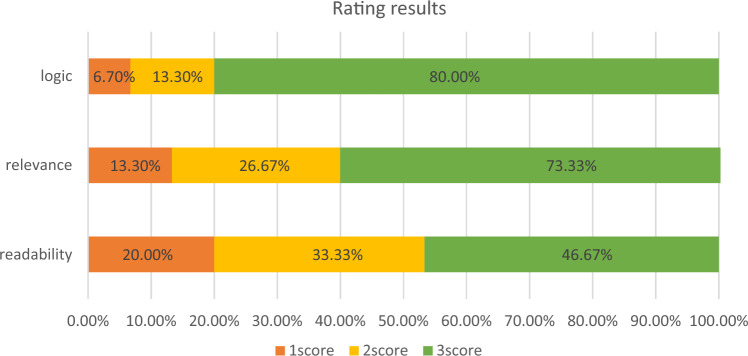
Overall reading effect test results.

The overall effectiveness verification results of the AI agent are shown in Fig. 7 These results demonstrate that the AI agent developed based on structured prompt language for performing entity-relationship extraction tasks in texts of reservoir dispatch regulations has been highly recognized by participants in terms of logical consistency, relevance, and readability. Over 80% of participants gave the highest scores for logical consistency, while less than 7% gave the lowest scores. Those who gave the lowest scores believed that the use of bullet points and various labels in the proposed method divided the overall content into multiple parts, making the sentences read disjointedly. However, removing the bullet points and labels would decrease the final extraction accuracy, as confirmed by the experimental results in Section "Baseline experiment setup". In terms of content relevance, over 73% of participants gave very high ratings, while the remaining 27% felt that the labels "persona" and "audience" were irrelevant to the extraction task. These two labels are designed to help the large language model better provide knowledge related to reservoir dispatching for text extraction. Removing them would introduce noise from other fields during the extraction process, affecting accuracy. Regarding readability, over 80% of participants gave high scores (including scores of 2 and 3). The remaining 20% found the hierarchical structure of the labels complex and difficult to understand, thinking that the design of the hierarchy leaned towards computer language, which could confuse those unfamiliar with computer knowledge. To address this, the design of this method considered this issue, and during implementation, indentation was specifically used to indicate the relationships and hierarchical structure between different labels. Based on the overall scoring results and the reasons given by participants for low scores, it can be concluded that the overall structure of the AI agent developed using structured prompt language has high logical consistency. The design closely revolves around the task of extracting entity relationships in reservoir dispatching procedures. The hierarchical structure is easy for most participants to understand. Overall, nearly half of the participants gave high ratings across the three evaluated aspects. Additionally, more than 30% of the participants who initially rated the readability as low (i.e., a score of 1) raised their readability score to 2 after reviewing content written using natural language prompts. These results indicate that the overall readability of the AI agent developed based on structured prompt language is good.

## Discussion

The method and AI agent developed in this paper have achieved good results in the task of reservoir dispatching entity relationship extraction, but there are still some deficiencies, mainly reflected in the following two aspects: First, the number of labels is limited. The current work has condensed eight types of labels based on the existing prompt patterns combined with the reservoir dispatching extraction task. However, as more and more engineering dispatching rules and increasingly complex watershed dispatching issues arise, it is necessary to further enrich the labels to meet the needs of the reservoir dispatching extraction task. Second, the extraction performance differences caused by different large language models of the AI agent. The AI agent developed in this paper allows users to choose from a variety of large language models. Due to the different training data and parameters obtained by each large language model, the performance will also vary, which in turn leads to different final extraction results. These factors are the key points for further testing and improvement in the future work of this paper.

## Conclusion and outlook

In the field of reservoir dispatch, there exist numerous complex engineering dispatch rules. Relying on models for extraction tasks consumes a lot of time and fails to meet the objective of promptly providing dispatch plans in the face of emergency disasters. Utilizing natural language prompts to guide large language models in completing extraction tasks presents two major challenges: cognitive burden and instability in model output. To address these issues, this paper proposes a novel idea for extracting entities and relationships from texts of reservoir dispatch regulations using structured prompt language, and designs an AI agent based on this method with the help of software engineering design principles. Compared to model extraction methods, this AI agent can quickly obtain structured triples, laying the foundation for promptly providing dispatch plans. Compared to methods that use natural language prompts for extraction, this AI agent has two main advantages: first, it includes a variety of labels. Through the study of existing prompt designs and then refining labels according to actual task scenarios, it can cover various important aspects of guiding large language models, For example, imposing relevant constraints on the large language model, explaining related specialized terms, providing case references, and then giving detailed task descriptions for each label greatly reduce the cognitive burden and impact of output instability caused by unclear expressions, non-standard writing, and the absence of restrictive conditions. Second, Organize labels and hierarchical structure definitions using BNF. BNF transforms the overall content into a structured format, facilitating the understanding by large language models and effectively improving the accuracy of entity and relationship extraction in dispatching texts.

Through benchmark experiments, the AI agent not only effectively reduced the cognitive burden and the impact of instability in model outputs but also achieved good results in the task of extracting entities and relationships from texts of reservoir dispatch regulations, with F1 scores for both entities and relationships extraction being above 80%. In the future, we will attempt to apply this new paradigm to the intelligent extraction of knowledge in various fields of water resources, promoting the intelligent development of water resource text information processing in the new stage. This method can also be applied to other fields. For example, in the medical field, it can quickly extract key information such as patient medical history, treatment plans, and drug reactions from a large number of medical texts, thereby improving diagnostic efficiency, research progress, and making more precise medical decisions. In the financial field, this method can extract company financial data, market trends, and investment risks from financial statements and market reports, facilitating better evaluation of investment opportunities and optimization of investment portfolios. In the customer service field, this method can extract customer complaints, purchase records, preferences, and interests to better understand user needs and provide personalized services. In the future, with continuous development and improvement of the technology, this method is expected to be applied in more fields, bringing new breakthroughs in information processing and decision support across various industries.

## Data Availability

Due to the confidentiality of the data used in this study, only a small part can be released. The data can be found at https://github.com/aichiroudemao/data1.
